# Biological Activities of Natural Products II

**DOI:** 10.3390/molecules27051519

**Published:** 2022-02-24

**Authors:** Halina Maria Ekiert, Agnieszka Szopa

**Affiliations:** Chair and Department of Pharmaceutical Botany, Faculty of Pharmacy, Jagiellonian University Medical College, 9 Medyczna Street, 30-688 Kraków, Poland

Natural products of different origin and their potential therapeutic activities are of unceasing widespread interest to many scientific teams from all around the world. The pharmaceutical, cosmetics, and health-food industries are constantly seeking novel solutions to offer customers attractive new biologically active natural products.

Theoretically, not only plants but also microorganisms, algae, fungi, lichens, and animals can be a rich source of natural products.

This Special Issue puts the spotlight most of all on higher plants. Only one publication focuses on lichens as an interesting yet still uncharted source of bioactive metabolites.

The greatest attention is still paid to natural products with anticancer, anti-inflammatory, and antioxidant properties. Both the total extracts and isolated individual compounds are investigated. Most articles have analyzed their mechanism of action at the cellular and/or molecular level.

The species studied belong to widely recognized medicinal plants utilized by official European medicine but also to plant species long known in traditional medicine of Middle, East, and Southeast Asia and South America, which still await discovery by official phytotherapy and/or cosmetology.

This Special Issue presents the results of research of scientific teams from different countries of the world in 12 original papers and two review articles.

Among the publications, of particular note are four papers (three original and one review) connected to plant biotechnology. They document the significance of biomass cultured in vitro as a potential rich source of bioactive secondary metabolites. These papers also highlight the possibility of control and stimulation of natural product biosynthesis in in vitro cultures and the feasibility of process scaling-up for commercial production in bioreactors.

We hope very much that the readers of *Molecules* will find this Special Issue interesting, stimulating, and of practical value.

Several original articles published in this Special Issue explore anticancer and anti-inflammatory activities of natural products; the results are presented below.

Delgado et al. [1] documented the antiproliferative and apoptotic activity of total hexane leaf extract of *Hymenaea courbaril*, a plant species known in traditional South American medicine as an anticancer agent, against the prostate adenocarcinoma PC-3 cell line. The authors identified the main active, safe, antiproliferative, apoptosis-inducing compound of the extract as caryophyllene oxide and documented its low toxicity to normal cells (lung fibroblasts MR-5).

Papierska et al. [2] presented the results of activity testing of two depsidones—physodic acid and salazinic acid of lichen origin—against colorectal cancer cell lines (two lines). These compounds have the ability to modulate signaling pathways (Nrf-2, NF-ĸβ, and STAT3 pathways). This modulation/intervention may provide a new approach to colon cancer prevention and/or treatment.

Kłeczek et al. [3] focused on extracts of blooming above-ground parts of *Carpesium divaricatum* (a plant species famous in traditional East Asian medicine) as a cytotoxic and anti-inflammatory agent. The results documented a moderate nonselective cytotoxic activity against the normal (prostate epithelial cells, keratinocites) and cancer cell lines (prostate cancer—2 lines, melanoma cells—2 lines, and osteosarcoma cells—2 lines). At sub-cytotoxic concentrations, these extracts showed a significant anti-inflammatory activity.

Li and Yang [4] were the first to carry out a comprehensive phytochemical analysis of phenolic compounds in the roots of *Platycodon grandiflorum*, which is a raw material known to be a rich source of triterpenoid saponins. Among 21 isolated compounds (lignoles, phenols, lignans, and one neolignan), two lignoles are new compounds discovered for the first time in the plant kingdom. The authors documented anti-inflammatory activity of all isolated compounds in the lipopolysaccharide (LPS)-stimulated murine macrophage cell model and their inhibitory effect on proinflammatory cytokins, IL-6, IL-12 p40, and TNFα.

Ko et al. [5] also concentrated their investigations on the anti-inflammatory activity of two prenyl-flavonoids—kuwanon T and sanggenon A—isolated previously by them from the root bark of *Morus alba*. This activity was documented on microglial cells and macrophages. Both compounds were proposed as potential candidates for the therapy and prevention of inflammatory diseases.

Gwon et al. [6] tested 6-shogaol, the main bioactive phenolic compound of dried ginger (*Zingiber officinale*, rhizome) with earlier-documented anti-inflammatory properties as a protectant against acute kidney injury (AKI), which is the side effect connected with cisplatin therapy (murine model). The results indicated that 6-shogaol exhibited therapeutic effects against AKI, e.g., via antioxidant and anti-inflammatory activity.

Ghosh et al. [7] evidenced the antimutagenic effect of *Morus alba* root bark ethanolic extract, showing its ability to prevent the nuclear damage caused by cyclophosphamide (CPP) during anticancer therapy (experimental animals—male rats). The authors proposed the combination therapy with CPP and plant-derived extracts for improvement of the prognosis and safety of therapy.

One article deals with other activities of plant extract, namely antidiarrheal and antibacterial actions.

Elmongy et al. [8] performed, for the first time, LC-ESI-MS/MS analysis of methanolic extract of roots of *Cupressus macrocarpa*, a famous ornamental and also medicinal plant with valuable essential oil in the leaves. Altogether, 39 compounds were identified (e.g., flavonoids, biflavonoids, polyflavonoids, catechins, and stilbenes); three of them were isolated for the first time. Antidiarrheal activity and antibacterial and antibiofilm effects of the extract and isolated compounds against *Salmonella enterica* (clinical isolate) were documented in vivo in mice. Rhamnoside of dihydrokaempferol was the most active compound.

The next article is an interesting proposal for cosmetic use.

Sabitov et al. [9] analyzed the phytochemicals in hydroalcoholic extracts of different organs of *Rosa platyacantha* wild-grown in the mountain area in Kazakhstan. The extract from closed flowers (flower buds) possessed the highest antioxidant activity (DPPH and ABTS tests) and the highest tyrosinase inhibitory potential. Additionally, this extract possessed collagenase inhibitory activity and cytotoxicity against the human melanoma cell line. The authors proposed the extract as a skin-lightening, anti-aging, and protective product for phytocosmetology.

Three original articles present the possibility of using different strategies known in plant biotechnology to obtain a potential rich source of bioactive natural products.

Thiem et al. [10], for the first time, worked out the micropropagation protocol for the rare European taxon characteristic of the Northern Hemisphere *Linnea borealis* L. var. *borealis*, which involved the development of numerous lateral buds from multi-node stem segments in agitated culture. The collection of in vitro propagated plantlets could be a good conservation strategy. The authors also established vital callus culture that could be a good source of biomass for a suspension rich in bioactive compounds (e.g., flavonoids, iridoids, saponins, and phenolics).

Another biotechnological strategy was evaluated by Klimek-Szczykutowicz et al. [11], who maintained bioreactor microshoot cultures of *Nasturtium officinale* (a rare, endangered aquatic plant species) with the addition of phenylalanine (Phe) and/or tryptophan (Trp) as biogenetic precursors of bioactive metabolites: polyphenolics, flavonoids, total and individual glucosinolates (GLs). Phe stimulated the production of GLs, antioxidant potential (CUPRAC and FRAP tests) and bacteriostatic activity of biomass extracts against bacterial strains causing skin diseases.

The results of similar experiments are presented by Kwiecień et al. [12], who grew the microshoots of *Scutellaria lateriflora*, a plant species of North-American origin, in agitated culture fed with phenylalanine (Phe) and tyrosine (Tyr) as biogenetic precursors of specific Scutellaria flavonoids and verbascoside, and additionally elicited with methyl jasmonate. The best strategy for total flavonoids and verbascoside was Phe-feeding. The authors documented a high antioxidant potential of the biomass extract using four different methods. Additionally, they established, for the first time, the bioreactor culture which produced high amounts of antioxidants.

Two review articles are dedicated to entirely different scientific problems. The review by Nowak-Perlak et al. [13] presents plant extracts and isolated individual phytochemicals, having the potential to be used in the fight against psoriasis, an autoimmune disease affecting 125 million people worldwide.

Motyka et al. [14] recapitulate the current status of *Salvia hispanica* (chia), a plant species of Middle- and South-American origin, in the European health-food industry. The current knowledge about the chemical composition, biological activities, application in the health food and cosmetics industries, and possible applications in phytotherapy and also biotechnological studies of this species are presented in this review.

Summing up, based on the excellent contributions to this issue, we are convinced that the readers of *Molecules* will find this second part of the Special Issue dedicated to biological activities of natural products very interesting and that the presented results of the investigations will be the inspiration for their own research in the near future.
